# Supplementary material for: Impaired Hippocampal Long-Term Potentiation and Memory Deficits upon Haploinsufficiency of MDGA1 Can Be Rescued by Acute Administration of D-Cycloserine
Source: Int J Mol Sci. 2024 Sep 6;25(17):9674. doi: 10.3390/ijms25179674 (PMC11394992; doi:10.3390/ijms25179674)
Supplement: Supplementary file 1 [file ijms-25-09674-s001.zip › ijms-3173205-supplementary.pdf]

Supplemental Information

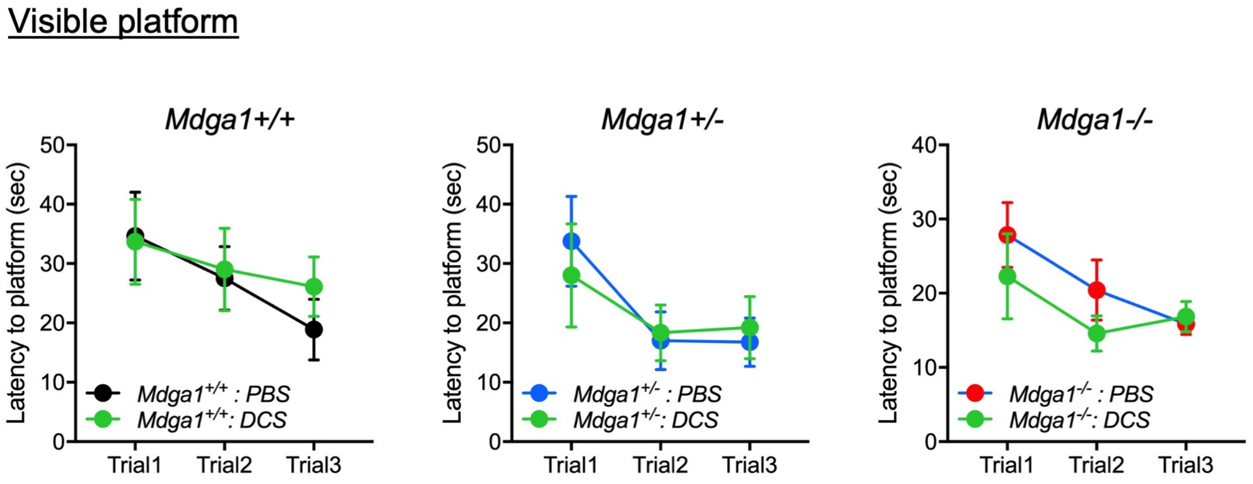

Figure S1
MDGA1 deficiency and D-cycloserine administration gave no significant difference in visible platform task. 2-way RM-ANOVA following Dunnet post hoc test.
Mdga1$^{+/+}$: PBS; n=8, D-cycloserine; n=9, medication $F_{1,15}$ = 0.153, p = 0.7014.
Mdga1$^{+/-}$: PBS; n=8, D-cycloserine; n=9, medication $F_{1,15}$ = 0.0141, p = 0.9072.
Mdga1$^{-/-}$: PBS; n=7, D-cycloserine; n=7, medication $F_{1,12}$ = 1.21, p = 0.2931.

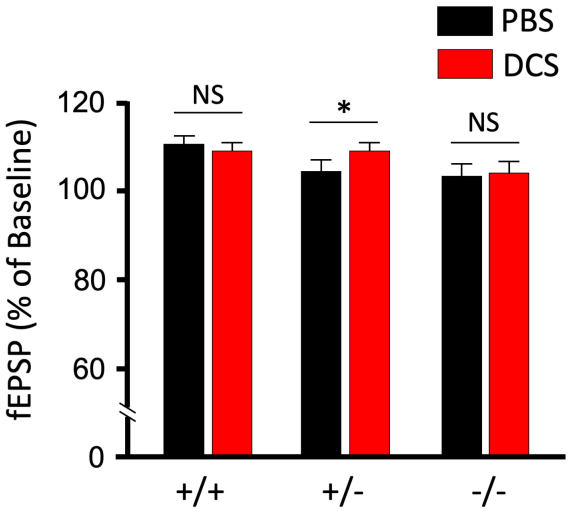

Figure S2
D-cycloserine significantly enhanced LTP in *Mdga1*$^{+/-}$ mice CA1 region. Later LTPs (60-80 min after stimulation) in the absence and presence of 10μM D-cycloserine were compared. Student's t-test. *Mdga1*$^{+/+}$: PBS; n=12, D-cycloserine; n=12. *Mdga1*$^{+/+}$: PBS; n=9, D-cycloserine; n=14. *Mdga1*$^{+/+}$: PBS; n=9, D-cycloserine; n=10. *P < 0.05.

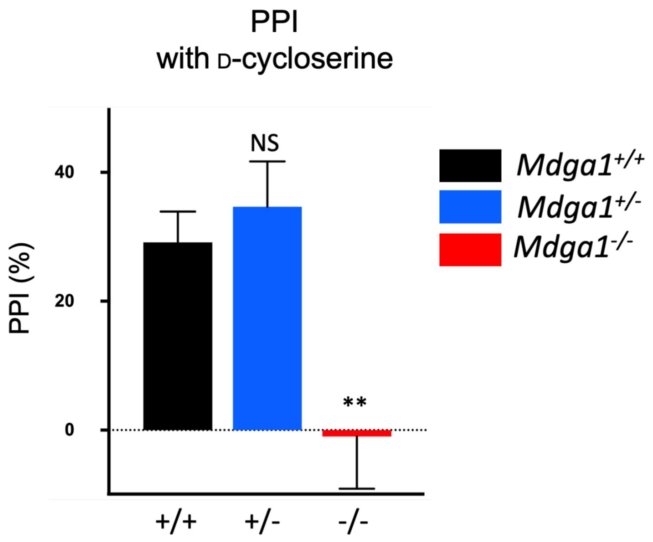

Figure S3
D-cycloserine administration did not ameliorate prepulse response of *Mdga1*$^{-/-}$ mice.
One-way ANOVA, following Dunnet post hoc test; genotype $F_{2,22} = 8.017$, $p = 0.0024$. **p<0.01 versus wild type mice. *Mdga1*$^{+/+}$ mice: n=9; *Mdga1*$^{+/-}$ mice: n=8; *Mdga1*$^{-/-}$ mice: n=8. Data are expressed as mean ± SEM.

Materials and Methods for Figure S3.

An LE 118–4 Startle and Fear Interface (Panlab, Barcelona, Spain) was used to measure the acoustic startle response of mice. One hour before the trial, 10mg/kg D-cycloserine was intraperitoneally injected in examined mice. The procedure was essentially the same as described previously [20]. Background noise was set at 60 dB. Pulse-alone trials consisted of a single white noise burst (120 dB, 40 msec). Prepulse + pulse trials consisted of a prepulse of noise (20 msec at 75 dB) followed 100 msec after prepulse onset by a startling pulse (120 dB, 40 msec). PPI of the startle response was calculated using the formula: %PPI = 100 - (startle response for prepulse and pulse trials/startle response for pulse-alone trials) × 100.
